# Altered Cytokine and BDNF Levels in Individuals with Autism Spectrum Disorders

**DOI:** 10.3390/brainsci12040460

**Published:** 2022-03-29

**Authors:** Yvonne M. Y. Han, Suk-Yu Yau, Melody M. Y. Chan, Chun-Kwok Wong, Agnes S. Chan

**Affiliations:** 1 Department of Rehabilitation Sciences, The Hong Kong Polytechnic University, Hong Kong SAR, China; sonata.yau@polyu.edu.hk (S.-Y.Y.); 18041801r@connect.polyu.hk (M.M.Y.C.); 2 Department of Chemical Pathology, Prince of Wales Hospital, The Chinese University of Hong Kong, Hong Kong SAR, China; ck-wong@cuhk.edu.hk; 3 Department of Psychology, The Chinese University of Hong Kong, Hong Kong SAR, China; aschan@cuhk.edu.hk

**Keywords:** immunologic function, autism, cognitive function, BDNF, biomarkers

## Abstract

Previous studies have shown that immunological factors are involved in the pathogenesis of autism spectrum disorders (ASDs). The present study examined whether immunological abnormalities are associated with cognitive and behavioral deficits in children with ASD and whether children with ASD show different immunological biomarkers and brain-derived neurotrophic factor BDNF levels than typically developing (TD) children. Sixteen children with TD and 18 children with ASD, aged 6–18 years, voluntarily participated in the study. Participants’ executive functions were measured using neuropsychological tests, and behavioral measures were measured using parent ratings. Immunological measures were assessed by measuring the participants’ blood serum levels of chemokine ligand 2 (CCL2) and chemokine ligand 5 (CCL5). Children with ASD showed greater deficits in cognitive functions as well as altered levels of immunological measures when compared to TD children, and their cognitive functions and behavioral deficits were significantly associated with increased CCL5 levels and decreased BDNF levels. These results provide evidence to support the notion that altered immune functions and neurotrophin deficiency are involved in the pathogenesis of ASD.

## 1. Introduction

Autism spectrum disorder (ASD) is a pervasive and lifelong developmental disorder. The prevalence of ASD is high, with recent statistics of 1 in 54 individuals being reported, making ASD one of the most prevalent of all childhood developmental disorders [1]. There is great phenotypic heterogeneity in ASD, ranging from severe mental retardation to isolated cognitive problems, such as stereotyped behavior and difficulty in understanding others’ feelings [2,3,4]. Although the causes of ASD are not well understood, increasing evidence has suggested that dysregulation of the immune system could play a role in the pathogenesis of ASD. Epidemiological studies have reported a greater family history of autoimmune disease in people with autism than in healthy controls [5]. Within individuals with ASD, a growing number of studies have reported altered profiles in various cellular and humoral immune subsets, as measured in both postmortem brains of ASD patients and peripheral blood circulation in ASD patients [6]. The suggested role of cytokines in autotoxicity has been supported through recent clinical studies [7,8], in which active neuroinflammatory processes with cytokine profiling and activation of microglia and astrocytes were found in the brains of patients with ASD, suggesting neuroimmune dysfunction in the brains of autistic individuals.

Among the various cytokines, subsets of proinflammatory chemokines appear to play a crucial role in mediating neuroinflammation found in individuals with ASD [9]. For example, postmortem brain and spinal cord samples from individuals with ASD showed heightened proinflammatory cytokines, including chemokine macrophage chemoattractant protein 1/chemokine ligand 2 (MCP-1/CCL2), compared to control specimens [7]. Marked increases in CCL2 and eotaxin were also found in the cerebrospinal fluid (CSF) of children with ASD [10]. In addition to the elevated neuroinflammatory response in the brains of individuals with ASD, ASD patients showed altered levels of circulating chemokines in the blood. For example, altered gene expression, elevated plasma CCL2 levels and elevated RANTES/CCL5 levels were observed in ASD patients. Notably, chemokine levels have been shown to correlate with the risk [11,12] and severity of behavioral abnormalities [13,14,15] of ASD. These findings suggest that the levels of chemokine production of CCL2 and CCL5 could be related to the severity of cognitive abnormalities in individuals with ASD, and these biomarkers can potentially serve as important tools for recognizing cognitive impairments in ASD patients.

Although research supports immunological involvement in the pathogenesis of ASD, there is relatively limited research investigating the relationship among altered immune systems and cognitive behavioral deficits in individuals with ASD. Some research suggests a mechanism through which the excessive activation of cytokines can lead to brain dysfunction related to disrupted production of growth factors and neurogenesis [16]. Brain-derived neurotrophic factor (BDNF) is a member of the “neurotrophin” family of growth factors and is known to have important functions in the formation, branching and connectivity of synaptic connections during development [17,18]. It also plays a pivotal role in synaptic plasticity and long-term potentiation and is critically involved in learning, memory, and higher-order thinking [19,20]. Pertinently, BDNF is found throughout the brain and in the periphery [21]. Circulating levels of BDNF have been shown to reflect BDNF levels in the brain [22,23]. Moreover, low levels of BDNF in the brain and serum have been reported in various neuropsychiatric disorders, including schizophrenia [24,25] and Alzheimer’s disease [26,27].

Additionally, recent studies have provided evidence to support the involvement of BDNF in autism through its neurotrophic effects on the developing brain [16,28]. This notion is supported by previous studies that have demonstrated aberrant synaptic development and disordered connectivity as an underlying cause of the behavioral characteristics observed in individuals with ASD [29,30]. However, the role of circulating BDNF remains unclear, and previous studies have shown inconsistent results. While some studies reported increased circulating levels of BDNF in patients with ASD [16,31,32], others showed lower circulating levels of BDNF in individuals in the ASD groups than in the typically developing (TD) controls [33]. Nevertheless, due to its essential role in neuronal development, altered levels of BDNF could explain the abnormal cortical connectivity reported in individuals with ASD [34].

Although it is largely unknown how immunologic factors specifically affect neural networks in the brains of individuals with ASD, a better understanding of the relationship between immunologic function, neurophysiology and cognitive function in people with ASD is highly significant. Establishing these relationships would provide a foundation for the causal mechanism and developmental course of neurodevelopmental disorders in ASD patients that could be used in future research. Given the evidence linking ASD with abnormal immunologic function, altered levels of BDNF, and impaired synaptic connectivity, we postulate that the variable severities of autistic features would be associated with impaired synaptic connectivity in the brains of autistic individuals; moreover, we hypothesize that dysregulation of neurotrophic factor function and increased proinflammatory responses would be the potential mechanisms. The present study investigated the role of immune dysfunction in children with ASD by examining the associations among immune responses, serum levels of BDNF, and severity of ASD symptoms. For this purpose, we investigated whether proinflammatory chemokines and altered BDNF levels play a role in the selective cognitive and behavioral symptoms of ASD by (1) examining the levels of the proinflammatory chemokines CCL2 and CCL5 and the levels of BDNF in the blood serum of children with ASD and then comparing these levels with those of TD children; (2) determining whether there was a correlation between chemokine (CCL2 and CCL5) and BDNF levels; and (3) determining whether proinflammatory chemokines and altered BDNF levels play a role in the selective cognitive and behavioral symptoms of people with ASD.

## 2. Materials and Methods

### 2.1. Participants

Twenty-one children with high-functioning ASD and 19 TD children aged between 6 and 17 years participated voluntarily in the study with their parents’ written informed consent. All of the children had an intelligence quotient (IQ) of 90, as measured by the short form of the Wechsler Intelligence Scale for Children–Fourth Edition, Hong Kong (WISC–IV HK [35]). Participants in the study were recruited by advertisements that were posted on our websites and invitation emails that were sent to parents in the contact database at the Neuropsychology Laboratory of the Chinese University of Hong Kong. All children were diagnosed with ASD based on the diagnostic criteria of the Diagnostic and Statistical Manual of Mental Disorders fifth edition (DSM-5) [36]. The diagnosis was confirmed by a clinical psychologist using the Autism Diagnostic Interview-Revised (ADI-R) [37], which is a standard clinical interview conducted with parents on the developmental history and current functioning of the children. TD children had no history of delayed developmental milestones or any neurological or psychiatric disorders as reported by their parents, did not meet the DSM–5 diagnostic criteria of ASD, and did not have ADI-R subscale scores over the specific cutoffs. Three children were excluded from the ASD group because two of them had the flu and a fever within 5 days of the blood test, and one child with ASD was prescribed immunosuppressive drugs. In addition, 16 children from the TD group were selected from a pool of 19 TD children to match the age (ASD: *M* = 10.28, *SD* = 3.39; TD: *M* = 11.36, *SD* = 3.61) and IQ (ASD: *M* = 102.56, *SD* = 7.82; TD: *M* = 105.06, *SD* = 10.24) of the individuals in the ASD group. Thus, 18 children with ASD and 16 TD children who were medication-free and in good physical health at the time of blood sample assays were included in the final analysis.

### 2.2. Procedures

All of the children and parents provided written informed consent before participating in the assessment and interviews. All children were individually administered the WISC-IV HK so that their IQs could be assessed, and a neuropsychological assessment of executive function was conducted that comprised five standardized tests: the D2 test of attention (D2) [38], the Children’s Color Trails Test (CCTT) [39], the Five Point Test (FPT) [40], the modified Wisconsin Card Sorting Test (WCST) [41], and the Rey–Osterrieth Complex Figure Test (Rey-O) [42]. Information on the children’s developmental and medical histories was collected through structured and individual interviews with parents. All of the assessments and interviews were administered by trained research assistants who were blinded to the rationale of the study and the group assignment.

Peripheral blood samples were collected from the children on separate days. A registered nurse drew 3 mL of blood mixed with ethylenediaminetetraacetic acid (EDTA) and 3 mL of clotted blood from each child using venipuncture at a medical clinic. The blood samples were kept in a thermally insulated bag and transported to the clinical laboratory where blood assays were performed. Blood sample processing and assays were performed by an experienced laboratory technician who was blinded to the clinical characteristics and group assignments of the participants. Each blood sample was centrifuged at 3000 rpm for 15 min, and the harvested serum was then stored at −80 °C for later processing. Serum levels of CCL2, CCL5 and BDNF were measured by enzyme-linked immunosorbent assay (ELISA) according to the manufacturer’s instructions. This study was conducted in accordance with the Helsinki Declaration of the World Medical Association Assembly. The research protocol was approved by the Joint Chinese University of Hong Kong—New Territories East Cluster Clinical Research Ethics Committee (Ref. No. 2013.520) and the Human Subjects Ethics Sub-Committee of the Hong Kong Polytechnic University (Ref. No. HSEARS20170316006).

### 2.3. Measures

#### 2.3.1. Immunological Measures

The concentrations of the chemokines CCL2 and CCL5 were assessed using BD™ human chemokine cytometric bead array (CBA) reagent (Becton Dickinson Biosciences Pharmingen, CA, USA). Samples were analyzed on a multifluorescence BD FACSCalibur™ flow cytometer using BD CellQuest™ and BD™ CBA software. Serum BDNF levels were measured using commercial ELISA kits purchased from Abcam (ab99978, Cambridge, UK).

#### 2.3.2. Behavioral Measures

*ADI-R.* The ADI-R [37] is an investigator-based interview for parents of children whose behaviors are relevant to the diagnosis of ASD or pervasive developmental disorder. The test results were scored in three separate domains: (1) reciprocal social interaction; (2) communication; and (3) restricted, repetitive, and stereotyped patterns of behaviors. All of these domains correspond to the DSM-V diagnostic criteria of ASD and were chosen to reflect the severity of their children’s core ASD symptoms, with higher scores indicating greater autistic features. Questions were asked concerning both the early developmental and current functioning of the child.

#### 2.3.3. Executive Functioning Measures

*WISC-IV HK short form.* The short form of the WISC-IV (HK) [35] is a psychometric measure that can be used to evaluate the general intellectual ability of children between 6 and 16 years old. The short form comprises two verbal subtests, Digit Span and Similarities, and two performance subtests, Matrix Reasoning and Coding. The measure yields a standard IQ score that has a mean of 100 and a standard deviation of 15.

*D2.* D2 [38] is a test of selective and sustained attention in which participants have to cancel out all target characters (a “d” that has two dashes placed above and/or below) and ignore other nontarget characters (a “d” or a “p” with more or less than two dashes above and/or below) within 20 s for each of the 14 trials. The concentration performance is calculated as the number of correctly cancelled target characters minus the total number of commission and omission errors demonstrated.

*CCTT.* The CCTT [39], which has a similar but simpler format than the traditional Trail Making Test, provides an evaluation of children’s attention, cognitive flexibility, and speeded visuomotor tracking ability without the influence of language. Color Trail 1 requires participants to quickly connect scattered numbers from 1 to 8 in ascending order, while in Color Trail 2, participants have to correctly connect scattered numbers from 1 to 15 that are concurrently embedded in alternate pink and yellow circles.

*FPT.* The FPT [40] measures figural fluency capacity and provides an evaluation of children’s cognitive flexibility. Participants are required to generate as many novel designs as possible by drawing straight lines to connect identically arranged dots presented in squares within 3 min.

*Rey-O.* The Rey-O [42] tests children’s visuospatial constructional functions, visuographic memory, and planning. Participants are presented with a geometric figure and some colored pencils and are asked to copy the design on a piece of paper, after which they are asked to recall the figure from memory following a delay period of 3 min.

*Modified WCST.* In the modified version [41], participants have to sort a series of cards according to two rules (i.e., the color or shape of each card). The participants received no information about the sorting rule when the cards were presented, but they were explicitly informed whether their choice was correct after each response. In each trial, three cards arranged in a pyramid were presented by E-prime (Psychology Software Tools, Inc. Pittsburgh, PA, USA) on a computer screen. The card at the top was the target card, and the two cards at the bottom were the choice cards. For half of the trials, the correct choice card was on the left, and for the other half of the trials, it was on the right. The participants had to match the target card with the correct choice card, based on the sorting rule at that moment, by pressing the left or right button on a button box that corresponded to the side of the choice card. There was no time limit for the participant to respond, and the stimuli stayed on the screen until the participant gave a response. There were a total of 5 test blocks with alternating sorting rules. A new block began after 7, 8, or 9 consecutive correct responses were made. The task was terminated if the criterion number of the consecutive correct responses was not met within 50 trials. A practice block that required 8 consecutive correct responses had to be completed before the 5 test blocks began.

### 2.4. Data Analyses

Independent sample *t*-tests were performed to compare the cognitive, behavioral, and immunological functioning of individuals in the ASD and TD groups. To reduce the number of statistical comparisons, one executive composite score was computed by summing and averaging the Z scores of the main dependent variables (DVs) of the five standardized neuropsychological tests. This method comprised D2 results, in which the main DV was the concentration performance; CCTT, in which the main DVs were the time to completion for Part 1 and Part 2; FPT, in which the main DV was the number of unique designs found in 3 min; and Rey-O, in which the main DV was immediate recall. For the modified WCST, the main DV was the mean number of total errors, which included both the number of perseverative errors and the failure to maintain sets. A low executive function composite score indicated poor performance. The relationship between the severity of autistic symptoms and executive function and between the concentrations of chemokines and BDNF was examined using Pearson’s correlations (two-tailed). Between-group comparisons of categorical variables were analyzed using chi-square tests. The likelihood ratio was reported for distributions that had more than 20% of cells with an expected count of less than 5. Because specific hypotheses were tested, the alpha level was unadjusted and set at 0.05 for all of the tests. All statistical analyses were performed using SPSS software (SPSS, Inc., Chicago, IL, USA).

## 3. Results

### 3.1. Demographic and Clinical Characteristics

Table 1 shows the demographic and clinical characteristics of the children. The ASD and TD groups were matched for age (*t* = 0.90, *p* = 0.38), sex (likelihood ratio = 3.37, *p* = 0.066) and intellectual functioning (*t* = 0.81, *p* = 0.43) as measured by WISC-IV HK. The ASD group demonstrated significantly more ASD-related symptoms than the TD group (t ranged from 6.93 to 11.05, ps < 0.001).

### 3.2. Deficient Executive Functioning in Children with ASD

Table 2 presents the means and standard deviations of the executive function composite score and the individual results in each of the five neuropsychological tests that were conducted.

An independent sample *t*-test showed that individuals in the ASD group demonstrated significantly lower executive function composite scores than individuals in the TD group. Specifically, individuals in the ASD group produced a significantly less unique design in the FPT (*t* = 3.70; *p* < 0.001) and committed a larger number of total errors (i.e., perseverative errors and failures to maintain sets) in the modified WCST (*t* = 2.51; *p* = 0.021) than individuals in the TD group. Individuals in the ASD group also tended to have worse concentration performance (*t* = 1.84; *p* = 0.075), take longer to complete the first trial of the CCTT (*t* = 1.88; *p* = 0.069), and have lower accuracy in the immediate recall of the Rey-O (*t* = 2.00; *p* = 0.054), although the group differences were only marginally significant. In sum, individuals in the ASD group had significantly poorer performance across the executive functioning tasks than their TD counterparts.

### 3.3. Altered Serum Concentrations of Chemokines and BDNF in Children with ASD

The results of an independent sample t-test showed that compared to individuals in the TD group, individuals in the ASD group had a significantly higher level of the inflammatory chemokine CCL5 (*t* = 2.86; *p* = 0.008) and a marginally significant trend of higher CCL2 levels (*t* = 2.00; *p* = 0.056) (Table 3). In addition, BDNF levels were lower in individuals in the ASD group than in individuals in the TD group (*t =* 2.31; *p* = 0.028).

### 3.4. Associations between the Severity of ASD Symptoms, the Executive Function Composite Score and the Levels of Chemokines and BDNF

Pearson’s correlation analysis was performed to assess the possible correlations between the behavioral symptoms of ASD as indicated by the three subscale scores of ADI-R, the executive function composite and the mean levels of the inflammatory chemokines CCL2 and CCL5, and BDNF. The results showed a significant correlation between the level of CCL5 and the social interaction subscale of the ADI-R and between the level of CCL5 and the communication subscale of the ADI-R; more specifically, a higher level of CCL5 was correlated with a higher score on the two subscales (*r* = 0.35, *p* = 0.047; *r* = 0.36; *p* = 0.041, respectively) (Table 4). Similarly, a significant correlation was found between the level of BDNF and each of the social interaction, communication and stereotyped behavior subscales of the ADI-R (*r* ranges from −0.39 to −0.49; *p* from 0.004 to 0.027). More specifically, the lower the level of BDNF was, the higher the three subscale scores of the ADI-R were. In addition, it was found that the executive function composite score was negatively correlated with the scores on the social interaction and communication subscales of the ADI-R (*r* = −0.36, *p* = 0.037; *r* = −0.58; *p* < 0.001, respectively).

## 4. Discussion

The present study examined cognitive dysfunction and behavioral deficits in high-functioning children with ASD and investigated whether these abnormalities were associated with altered proinflammatory chemokine and BDNF levels. When compared to age-, IQ- and sex-matched TD individuals, individuals with ASD showed significantly more severe social communication and interaction deficits with more restrictive repetitive behaviors, weaker executive functioning performance, higher mean levels of the proinflammatory chemokine CCL5, and lower mean levels of blood serum BDNF. Importantly, a higher CCL5 level was found to be associated with greater deficits in social interaction and communication, and a lower serum BDNF level was found to be associated with a higher severity across all ASD core behavioral symptoms.

Consistent with previous studies that included individuals with ASD who were of a younger [11,13] or older [43] age when compared to this study sample, we revealed an elevated proinflammatory chemokine profile in individuals with ASD. Specifically, CCL5 levels were found to be significantly higher, and the trend of higher levels of CCL2 was found to be marginally significant in our sample. Importantly, this research pioneered the establishment of a significant positive association between CCL5 and ASD social deficits measured by the ADI-R, the ‘gold standard’ [44,45] in ASD diagnostic evaluation. This association implies that immune dysfunction could be a possible mechanism underlying the manifestation of ASD core behavioral symptoms. Indeed, our current results are supported by previous animal studies showing that reducing proinflammatory chemokines with the administration of resveratrol [46] could result in a reduction in ASD behavioral phenotypes [47,48]. Notably, the mechanisms of how immune dysfunction results in ASD behavioral deficits remain to be investigated, and abnormal spinogenesis associated with cytokine and chemokine dysregulation [49] might be a possible candidate for further studies. For instance, converging data from postmortem brain tissues of individuals with ASD [50,51] and studies using animal models of ASD [52,53] have suggested an increase in the number of immature dendritic spines. In addition, the abnormal turnover and structure of dendritic spines have been observed in individuals with ASD that could consequently contribute to delayed spine maturation (see Phillips and Pozzo-Miller [54] for a review). Furthermore, abnormalities in spine dynamics have been shown to result in selective cognitive and behavioral symptoms with neural circuit dysfunction (i.e., disordered synaptic connectivity) in individuals with ASD [55]. Future research that investigates the causal relationship between proinflammatory cytokines and immature spinogenesis in ASD animal models, as well as the association between aberrant immune function and functional connectivity in human ASD subjects, would be beneficial to improve our understanding of the pathophysiology of this neurodevelopmental disorder.

A recent review concluded that significantly reduced BDNF expression is commonly found in patients with neuropsychiatric disorders [56]. In line with this observation, as well as being consistent with some previous studies [12,57], our data showed that children with ASD had a lower level of serum BDNF than TD individuals. Furthermore, a lower BDNF level was found to be correlated with a greater severity in all core ASD behavioral symptoms (i.e., social communication/interaction deficits and restricted repetitive behaviors) reflected in the ADI-R scores, suggesting the importance of BDNF abnormalities in the manifestation of ASD symptomatology. BDNF is known to play a crucial role in modulating structural and synaptic plasticity in the brain [58,59]. Notably, enhancing BDNF concentration has been found to promote dendritic [60] and axonal [61] growth in an activity-dependent manner [62]. Additionally, in ASD human and animal studies, a generalized reduction in the size and number of dendrites has been observed [63]; moreover, reduced axonal diameter and white matter in brain regions related to social cognition and information processing hubs have been found using diffusion tensor imaging techniques in ASD patients [64,65]. Based on these findings, together with our data, we postulate that BDNF reduction in individuals with ASD might be associated with dendritic and axonal abnormalities in these individuals, although this concept has to be further verified in future studies. It is worth pointing out that our results are in contrast with two studies with a Chinese population showing that BDNF is higher, instead of lower, in individuals with ASD than in TD individuals [66,67]. This outcome could be attributed to the fact that the other studies recruited ASD participants who were younger in age (mean age = approximately 4 years) than our sample (mean age = 10.28 years). This is consistent with previous meta-analysis reporting higher BDNF levels found only in children, but not adults with ASD [67]. Additionally, previous studies have also shown that BDNF levels decrease with increasing age in healthy humans [68]. A higher BDNF level in younger ASD participants and a lower BDNF level in older ASD participants might imply that these individuals differ in the developmental trajectory of BDNF levels that might modulate cognitive development, which warrants further research.

Social communication is a highly complex construct that requires an individual to process and integrate relevant information from the environment to produce adaptive behaviors for social interaction [69]. The successful integration and the manipulation of social information require a person to have multiple intact executive functions, such as cognitive flexibility and attention, which involve coordination between primarily the frontal lobe and other brain regions [70]. Importantly, both cognitive flexibility [71] and attention [72] have been found to be associated with social communication skills. Impairments in these cognitive processes have been hypothesized to underlie social communication dysfunction, which is a core symptom in people with ASD regardless of their level of functioning [73]. Indeed, our results showed that ASD individuals had weaker executive functioning, specifically in cognitive flexibility (indicated by statistically poorer performance on the FPT and modified WCST), which is consistent with results from a meta-analysis [74]. We further showed that poorer executive functioning was associated with poorer social interaction/communication skills (indexed by higher ADI-R subscores in these domains), which reiterated the importance of intact executive functioning in social information processing. Increasing evidence supports the notion that the executive functioning deficits in individuals with ASD are due to underlying brain abnormalities that affect the connection of neural networks. For instance, previous human studies have reported disordered long-distance corticocortical connectivity and localized connectivity in the brains of individuals with ASD when they perform various cognitive tasks, including cognitive flexibility [75]; in addition, disordered connectivity has been shown to be correlated with the severity of impairment in social communication and of restrictive/repetitive behaviors [76]. Given that connections of the neuronal circuit have been shown to be mediated by the remodeling of dendrites and that this remodeling process has been associated with changes in BDNF and cytokine levels in the brain, as discussed above, we hypothesized that BDNF expression, chemokine concentration and executive functioning are interrelated. Surprisingly, although BDNF and chemokine levels, as well as executive functioning, were indeed abnormal in individuals with ASD, as shown and discussed above, the correlations of BDNF levels and executive functioning composite scores (*p* = 0.057), CCL5 levels and executive functioning composite scores (*p* = 0.138), and CCL5 and BDNF levels (*p* = 0.366) did not reach statistical significance. However, our results could be limited by a relatively small sample size, or they could also be explained by the inherent heterogeneous clinical and neurobiological profiles of individuals with ASD [77]. Furthermore, it remains unclear what causes the changes of CCL5 and BDNF levels over time in children with ASD. Future research with a larger sample size, a more homogenous profile of individuals, and other age cohorts might be helpful in exploring these relationships to strengthen our understanding of neuroinflammation, neuronal modeling and executive functioning in individuals with ASD.

## 5. Conclusions

This study aimed to investigate the relationships between immunological abnormalities, cognitive deficits and behavioral manifestations in children with ASD. When comparing the results from 16 TD and 18 ASD individuals aged 6–18 years, ASD individuals exhibited greater deficits in cognitive functions along with altered levels of immunological measures. Importantly, aberrant cognitive functions and associated behavioral deficits in individuals with ASD were significantly associated with heightened CCL5 levels and lower BDNF levels. Our results support the notion that abnormal immune functions are associated with neuronal deficits, are indexed by decreased levels of BDNF, and manifest as selective cognitive and behavioral symptoms in individuals with ASD.

## Figures and Tables

**Table 1:** Demographic and clinical characteristics of children with autism (ASD) and the typically developing (TD) controls.

Characteristics	ASD (n = 18)	TD (n = 16)	*t* or χ^2^	*p*
Age	10.28 (3.39)	11.36 (3.61)	0.90	0.38
IQ	102.56 (7.82)	105.06 (10.24)	0.81	0.43
Gender (Male %) ^	61.5%	38.5%	3.37	0.066
ADI-R social interaction	19.17 (7.22)	4.19 (3.43)	7.86	<0.001 ***
ADI-R communication	13.67 (4.61)	1.25 (1.13)	11.05	<0.001 ***
ADI-R stereotyped behavior	5.17 (2.28)	0.25 (0.77)	8.60	<0.001 ***

Note. Data are presented as the means (SD); IQ, intelligence quotient as assessed by the Chinese version of Wechsler Intelligence Scale for Children Fourth Edition; ADI-R, Autism Diagnostic Interview-Revised; SRS-2, Social Responsiveness Scale Second Edition; ^ Likelihood ratio Chi-square tests were performed for distribution violating the sample size assumption of Chi-square test. *** *p* < 0.001.

**Table 2:** Comparison of mean scores in executive function tasks between children with autism (ASD) and typically developing (TD) controls.

	ASD (n = 18)	TD (n = 16)	*t*	*p*
Executive Function Composite	−5.65 (7.32)	0.34 (4.68)	2.80	0.009 **
D2 Test of Attention				
Concentration performance	124.50 (43.04)	151.75 (43.88)	1.84	0.075
Children’s Color Trails Test				
Trial 1–Time to completion (seconds)	29.50 (12.34)	22.31 (9.59)	1.88	0.069
Trial 2–Time to completion (seconds)	58.72 (24.17)	49.56 (18.98)	1.22	0.23
Five Point Test				
Number of unique designs in 3 min	20.78 (6.66)	31.31 (9.82)	3.70	0.001 **
Rey–Osterrieth Complex Figure Test				
Immediate recall	10.36 (5.16)	14.66 (7.31)	2.00	0.054
Modified Wisconsin Card Sorting Test				
Mean number of total errors	3.00 (3.13)	1.10 (0.68)	2.51	0.021 *

Note. Data are presented as the means (SD). ** *p* < 0.01; * *p* < 0.05.

**Table 3:** Comparison of mean concentrations of chemokines and neurotrophin in children with autism (ASD) and typically developing (TD) controls.

	ASD (n = 18)	TD (n = 15 ^)	*t*	*p*
**Chemokines**				
CCL2 (pg/mL)	181.10 (68.18)	143.17 (37.69)	2.00	0.056
CCL5 (pg/mL)	43,867.64 (15,124.57)	31,070.68 (8103.60)	2.86	0.008 **
**Neurotrophin**				
BDNF (pg/mL)	12.08 (5.02)	15.94 (4.21)	2.31	0.028 *

Note. Data are presented as the means (SD). ^ One datum is missing from the TD group. ** *p* < 0.01; * *p* < 0.05.

**Table 4:** Pearson association analysis of the three subscales of ADI-R with executive function composite, mean concentrations of CCL2, CCL5, and BDNF in all participants enrolled (n = 33 ^).

	ADI-R Social Interaction	ADI-R Communication	ADI-R Stereotyped Behavior	Executive Function Composite	CCL2	CCL5	BDNF
ADI-R social interaction	1.00						
ADI-R communication	0.90 ***	1.00					
ADI-R stereotyped behavior	0.75 ***	0.78 ***	1.00				
Executive Function Composite	−0.36 *	−0.58 ***	−0.28	1.00			
CCL2	0.144	0.20	0.10	−0.09	1.00		
CCL5	0.35 *	0.36 *	0.28	−0.26	0.29	1.00	
BDNF	−0.49 **	−0.47 **	−0.39 *	0.33	0.08	−0.16	1.00

Note. ADI-R, Autism Diagnostic Interview-Revised. ^ One datum is missing from the TD group. *** *p* < 0.001; ** *p* < 0.01; * *p* < 0.05.
